# Supplementary material for: Patient exposure in the basic science classroom enhances differential diagnosis formation and clinical decision-making
Source: PeerJ. 2015 Feb 26;3:e809. doi: 10.7717/peerj.809 (PMC4349165; doi:10.7717/peerj.809)
Supplement: Supplementary file 1 [file peerj-03-809-s001.docx]

## Question 5: Opinion Scale/Likert

Patient encounters should be incorporated into the Pathology Block next year.

| | Percent Answered |
|---|---|
| Strongly Disagree | 6.383% |
| Disagree | 8.511% |
| Neither Agree nor Disagree | 12.766% |
| Agree | 36.17% |
| Strongly Agree | 36.17% |
| *Unanswered* | 0% |

## Question 6: Opinion Scale/Likert

More patient encounters should be included in the first-year courses.

| | Percent Answered |
|---|---|
| Strongly Disagree | 4.255% |
| Disagree | 12.766% |
| Neither Agree nor Disagree | 17.021% |
| Agree | 34.043% |
| Strongly Agree | 31.915% |
| *Unanswered* | 0% |

## Question 7: Opinion Scale/Likert

The DDX assignments associated with the patient encounters helped me develop differential diagnosis formation skills.

| | Percent Answered |
|---|---|
| Strongly Disagree | 4.255% |
| Disagree | 19.149% |
| Neither Agree nor Disagree | 17.021% |
| Agree | 31.915% |
| Strongly Agree | 25.532% |
| *Unanswered* | 2.128% |

### Question 8: Opinion Scale/Likert

The DDX assignments associated with the patient encounters helped me develop clinical decision-making skills (i.e. questions to ask, tests to order, treatments to prescribe, etc.).

| | Percent Answered |
|---|---|
| Strongly Disagree | 4.255% |
| Disagree | 10.638% |
| Neither Agree nor Disagree | 10.638% |
| Agree | 57.447% |
| Strongly Agree | 17.021% |
| *Unanswered* | 0% |

### Question 9: Opinion Scale/Likert

I have a better understanding of pathology through the patient encounter experiences.

| | Percent Answered |
|---|---|
| Strongly Disagree | 8.511% |
| Disagree | 17.021% |
| Neither Agree nor Disagree | 21.277% |
| Agree | 38.298% |
| Strongly Agree | 14.894% |
| *Unanswered* | 0% |

### Question 10: Opinion Scale/Likert

The patient encounters helped me to better link a patient's pathology with a patient's clincial presentation.

| | Percent Answered |
|---|---|
| Strongly Disagree | 4.255% |
| Disagree | 10.638% |
| Neither Agree nor Disagree | 14.894% |
| Agree | 44.681% |
| Strongly Agree | 23.404% |
| *Unanswered* | 2.128% |

### Question 11: Opinion Scale/Likert

The patient encounters helped me to better recognize key elements of clinical history and exam in a patient.

| | Percent Answered |
|---|---|

| | |
|---|---|
| Disagree | 10.638% |
| Neither Agree nor Disagree | 23.404% |
| Agree | 44.681% |
| Strongly Agree | 21.277% |
| *Unanswered* | 0% |

### Question 12: Opinion Scale/Likert

The patient encounters improved my confidence to interact with patients.

| | Percent Answered |
|---|---|
| Strongly Disagree | 6.383% |
| Disagree | 34.043% |
| Neither Agree nor Disagree | 31.915% |
| Agree | 21.277% |
| Strongly Agree | 6.383% |
| *Unanswered* | 0% |

### Question 13: Opinion Scale/Likert

The patient encounters gave me a better sense of what questions would be important to ask patients.

| | Percent Answered |
|---|---|
| Strongly Disagree | 2.128% |
| Disagree | 10.638% |
| Neither Agree nor Disagree | 12.766% |
| Agree | 59.574% |
| Strongly Agree | 14.894% |
| *Unanswered* | 0% |

### Question 14: Opinion Scale/Likert

The patient encounters gave me a better sense of what diagnostic procedures or tests to order for patients.

| | Percent Answered |
|---|---|
| Strongly Disagree | 4.255% |
| Disagree | 25.532% |
| Neither Agree nor Disagree | 6.383% |
| Agree | 48.936% |
| Strongly Agree | 14.894% |
| *Unanswered* | 0% |

The patient encounters gave me a better appreciation of the social and emotional challenges that patients go through.

| | Percent Answered |
|---|---|
| Strongly Disagree | 2.128% |
| Disagree | 2.128% |
| Neither Agree nor Disagree | 8.511% |
| Agree | 44.681% |
| Strongly Agree | 42.553% |
| *Unanswered* | 0% |

## Question 16: Opinion Scale/Likert

The patient encounters helped to give you insight into what your clinical experience might be like.

| | Percent Answered |
|---|---|
| Strongly Disagree | 4.255% |
| Disagree | 6.383% |
| Neither Agree nor Disagree | 19.149% |
| Agree | 51.064% |
| Strongly Agree | 17.021% |
| *Unanswered* | 2.128% |
